# S180. MICROSTRUCTURE COMPLEXITY OF THE THALAMUS IN SCHIZOPHRENIA: A NODDI STUDY

**DOI:** 10.1093/schbul/sby018.967

**Published:** 2018-04-01

**Authors:** Yasser Alemán-Gómez, Timo Roine, Elena Najdenovska, Philippe Golay, Zita Rovó, Raoul Jenni, Martine Cleusix, Philippe Conus, Kim Q Do Do, Pascal Steullet, Meritxell Bach Cuadra, Philipp Baumann

**Affiliations:** 1 Centre Hospitalier Universitaire Vaudois (CHUV) and University of Lausanne (UNIL); 2 DP-CHUV (TIPP)

## Abstract

**Background:**

Schizophrenia is a neurodevelopmental disease arising from complex interactions between genetic and environmental factors that cause disconnectivity within core brain networks including the thalamus. The thalamus has a central role in the pathophysiology of schizophrenia, however to what extent and how it is affected at the microstructural level is still a matter of debate. In the current study, we apply the Neurite Orientation Dispersion and Density Imaging (NODDI) [1], a recently developed MRI technique, which allows the estimation of the microstructural complexity of dendrites and axons in vivo.

**Methods:**

Twenty-three patients with schizophrenia (SCHZ) were recruited from the Service of General Psychiatry (Lausanne University Hospital, Switzerland) (40.18 ± 9.2yo; 18/5 males/females) and 27 healthy controls (HC) (37.7 ± 7.95yo; 18/9 males/females). Magnetization-Prepared Rapid Acquisition Gradient Echo (MPRAGE) and a diffusion spectrum imaging (DSI) was performed on a 3-Tesla scanner (MAGNETOM Trio a Tim system, Siemens, Germany). Thalamus segmentation was performed on the MPRAGE sequence with an in house-pipeline using Freesurfer v5.0.0 for segmentation which was then refined to remove voxels within the ventricles and/or overlapping the internal capsule [2]. Orientation Dispersion Index (ODI), Intracellular Volume Fraction (ICVF) and, Isotropic Volume Fraction (ISOVF) were estimated based on the DSI sequence with NODDI [1]. General Linear Models (GLM) were estimated with outcome measures (ICVF, ISOVF, ODI) as dependent variables, group membership as a fixed factor (HC vs. SCHZ) and age and gender as potential covariates.

**References:**

1Battistella G, Najdenovska E, Maeder P, et al. Robust thalamic nuclei segmentation method based on local diffusion magnetic resonance properties. Brain Struct Funct 2016. DOI:10.1007/s00429-016-1336-4.

2Zhang H, Schneider T, Wheeler-kingshott CA, Alexander DC. NeuroImage NODDI : Practical in vivo neurite orientation dispersion and density imaging of the human brain. Neuroimage 2012; 61: 1000–16.

**Results:**

Mean ODI was significantly increased in schizophrenia patients compared to controls in the right thalamus (F(1,48)=5.032, p= .030, np2 = .095) and in the left thalamus (F(1,48)=4.500, p= .039, np2 = .086). When controlled for age and gender, the difference remained significant for the right thalamus (F(1, 46) = 4.197, p = .046, np2 = .084) but reduced to trend level for the left thalamus (F(1, 46) = 4.029, p = .051, np2 = .081). There were no significant differences on the other measures (ICVF, ISOVF).

**Discussion:**

Our results show that the thalamus is affected in patients with SCHZ at the microstructural level. The observed increase in ODI, which estimates the dispersion of neurite orientations, suggests disrupted neurite organization in patients as compared to HC.

